# How to Turn a Genetic Circuit into a Synthetic Tunable Oscillator, or a Bistable Switch

**DOI:** 10.1371/journal.pone.0008083

**Published:** 2009-12-07

**Authors:** Lucia Marucci, David A. W. Barton, Irene Cantone, Maria Aurelia Ricci, Maria Pia Cosma, Stefania Santini, Diego di Bernardo, Mario di Bernardo

**Affiliations:** 1 Telethon Institute of Genetics and Medicine (TIGEM), Naples, Italy; 2 Department of Computer and Systems Engineering, Federico II University, Naples, Italy; 3 Bristol Centre for Applied Nonlinear Mathematics, University of Bristol, Bristol, United Kingdom; 4 MRC Clinical Sciences Centre Faculty of Medicine, Imperial College London, London, United Kingdom; IBM Thomas J. Watson Research Center, United States of America

## Abstract

Systems and Synthetic Biology use computational models of biological pathways in order to study *in silico* the behaviour of biological pathways. Mathematical models allow to verify biological hypotheses and to predict new possible dynamical behaviours. Here we use the tools of non-linear analysis to understand how to change the dynamics of the genes composing a novel synthetic network recently constructed in the yeast *Saccharomyces cerevisiae* for In-vivo Reverse-engineering and Modelling Assessment (IRMA). Guided by previous theoretical results that make the dynamics of a biological network depend on its topological properties, through the use of simulation and continuation techniques, we found that the network can be easily turned into a robust and tunable synthetic oscillator or a bistable switch. Our results provide guidelines to properly re-engineering *in vivo* the network in order to tune its dynamics.

## Introduction

The emerging field of Synthetic Biology aims at constructing novel biological circuits in the cell [1]. It uses quantitative mathematical models in order to design and implement new cellular functions in a predictable and reliable fashion. In [2], we described the construction of a novel synthetic network in *Saccharomyces cerevisiae*, IRMA, built for *In vivo* Reverse-engineering and Modelling techniques Assessment. We measured time series and steady-state expression data after multiple perturbations and used them to assess the state of the art of both modelling and reverse-engineering techniques. The yeast synthetic network is composed of five genes that directly regulate the transcription of each other (*CBF1*, *GAL4*, *SWI5*, *GAL80* and *ASH1*) and includes one protein-protein interaction (Gal4-Gal80). The topology (Figure 1 in [2]) consists of one transcriptional positive feedback loop containing a delayed interaction (with a fixed time delay of 100 minutes) and two negative feedback loops. The first negative loop consists of direct transcriptional interactions between genes (*CBF1*, *GAL4*, *SWI5* and *ASH1*), while the second is present only in glucose growing condition, when there is a protein-protein interaction between Gal4 and Gal80. Such negative loop can be switched off by culturing cells in galactose. We derived and identified a non-linear Delay Differential Equations model containing 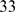 parameters (Supplemental Data in [2]). Our formalism uses non-linear Hill functions to describe transcriptional interactions and a phenomenological law to describe the protein-protein interaction triggered by the input. The model provides a reasonable compromise between accuracy and simplicity.

**Figure 1:**
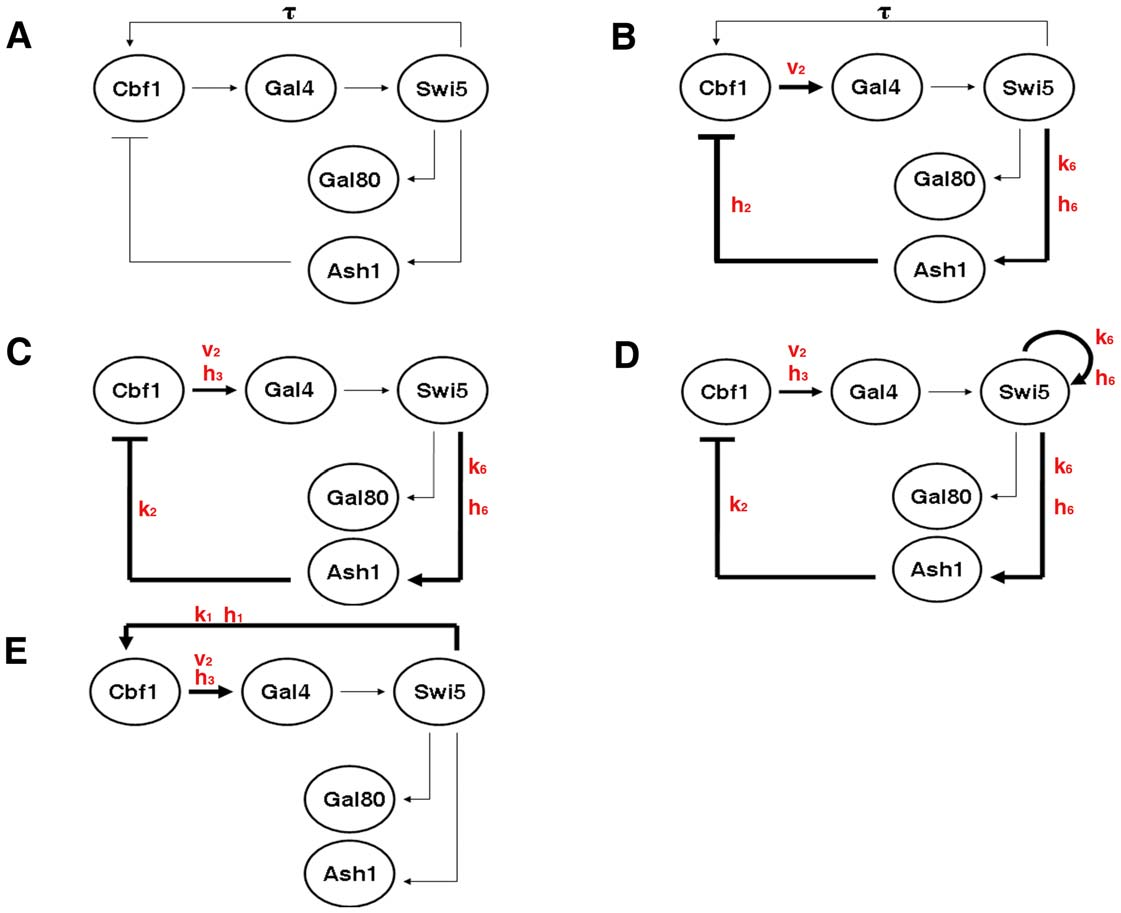
Re-engineering the topology in order to turn IRMA into an oscillator or a switch. Comparison between the topology of the actual version of the network (A) and the re-engineered topologies (B)–(E); in all the cases we only consider the galactose growing condition. A thicker line corresponds to an increase of the strength of the corresponding interaction; the strength is intended in terms of Michaelis-Menten coefficient and/or Hill coefficient and/or maximal transcriptional velocity. The parameters in red are the ones that we are varying from the nominal value. (A) Topology of IRMA. In galactose growing conditions, the topology consists of one delayed positive and one negative feedback loop, since the protein-protein interaction between Gal4 and Gal80 is switched off. (B) Re-engineering of IRMA in order to turn it into an autonomous oscillator, Scenario 1. Tuning the parameters v_2_, k_6_, h_2_ and h_6_ we increase the strength of the following interactions: Cbf1 on Gal4, Swi5 on Ash1 and Ash1 on Cbf1. Both the original positive and the negative feedback lops are present. (C) Re-engineering of IRMA in order to turn it into an autonomous oscillator, Scenario 2. Tuning the parameters v_2_, k_1_, k_2_, k_6_, h_3_ and h_6_ we increase the strength of the following interactions: Cbf1 on Gal4, Swi5 on Ash1 and Ash1 on Cbf1. The original positive feedback loop is removed. (D) Re-engineering of IRMA in order to turn it into an autonomous oscillator, Scenario 3. The topology is identical to the one in Scenario 2 with the addition of a positive auto-feedback-loop on Swi5. The tuned parameters are: v_2_, k_1_, k_2_, k_6_, h_3_ and h_6_. (E) Re-engineering of IRMA in order to turn it into a bistable switch, Scenario 4. Properly tuning the parameters v_2_, k_1_, k_2_, h_1_ and h_3_ we increase the strength of the following interactions: Cbf1 on Gal4, Swi5 on Cbf1. The negative feedback loop is removed.

The main aim of this paper is to show how to use novel tools from numerical bifurcation theory (e.g. DDE-BIFTOOL [3], able to deal with delayed systems), together with recent results on the link between the dynamics and topology of networks, in order to redesign a synthetic circuit. The need to modify a synthetic network after its biological implementation is common practice in Synthetic Biology. When a novel network is built, e.g. a synthetic oscillator, the design at the very beginning is often difficult and can lead to misleading results mainly due to the lack of quantitative characterisation of network components [4]. In our model-supported approach, the analysis of the previously identified mathematical model allows to increase the predictability of the network dynamics and experimental re-engineering, decreasing the amount of *in vivo* experiments and *post hoc* tweaking to be performed [4], [5]. The model predictions are used to determine how to tune the system parameters, and hence their physical counterparts, in order to change the dynamic behaviour of the network. Of note, the use of bifurcation theory for classification and categorization of the dynamics of species in a reaction mechanism, initiated in [6], is now commonly adopted for the construction and fine-tuning of synthetic networks (see [7] for an overview).

In particular, the aim is to understand if and how IRMA can be turned into a robust and tunable synthetic oscillator or a bistable switch. Oscillations have a crucial role in cell behaviour: the circadian clock and the cell cycle are common examples [8]. Currently, the interest of many researchers is focused on the properties of cellular oscillations that only depend on the topology of the reaction network, transcending the individual species involved [9]–[11].

In the case of IRMA, the goal is challenging, both in terms of the mathematical analysis and in terms of the *in vivo* implementation. Up to now, only small topologies have been analyzed, and the synthetic oscillators experimentally built consist of a few genes (e.g. [12]–[16]). Moreover, to our knowledge, numerical continuation techniques for DDEs model have not been applied to the analysis of synthetic gene networks up to now. We found that multi-step processing of gene products in the negative feedback loop and strong cooperativity in gene regulation are the ingredients to elicit robust oscillations.

In addition, we discovered that by reducing the topology of the network to a single positive feedback loop, IRMA can be turned into a bistable system (a “toggle switch”, that toggles between two discrete, alternative stable steady states). Hysteretic examples have been observed in several natural examples, including the control of lactose utilization in *E. coli*, and ensuring unidirectional cell-cycle progression in eukaryotes [17]. Synthetic switches have been built both in bacterial [18] and mammalian [19] cells for a variety of applications (e.g. gene therapy, construction of bio-sensors and research tools).

## Results

### Turning IRMA into an Oscillator

In [2], in order to analyze the dynamic behavior of the network, we performed perturbation experiments by shifting cells from glucose to galactose (switch-on experiments, used to fit the kinetic parameters), and from galactose to glucose (switch-off experiments, used to test the model predictive performance). Experimental data and the model simulated data (Figure 3 in [2]) show a clear delay in the dynamics of *CBF1* both in the switch-on and in the switch-off time-series. Regarding the transient dynamics of the switch-on, we observed seemingly damped oscillatory behavior in *SWI5* and *CBF1* concentrations and monotonic dynamics for all the other genes. In the switch-off, as expected, the transcription of the network genes is rapidly and monotonically turned off.

With the aim of tuning the dynamics of IRMA and turning it into an autonomous biochemical oscillator, we shall seek to achieve the desired dynamic behaviour by appropriately varying the model parameters. In so doing it is obviously fundamental both to remain inside the physically feasible range and to minimize the number of changes to the existing network topology and nominal parameter values, in order to speed up the experimental implementation.

In our specific case, the number of physical parameters is quite high (33), thus an exhaustive exploration of the parameter space would be excessively complicated and time consuming. On the other hand, from the analytical view point it is cumbersome to get any results about the structural stability of equilibria under parameters variations since the system is time-delayed and highly non-linear, due to the large value that the Hill coefficients can assume. For the case of our multi-parametric delayed gene network, it is then crucial to restrict the number of parameters to be changed to induce sustained oscillations. For the selection of the parameter subset to be used to carry out the bifurcation analysis, we use as guidelines the links between the topology and the occurrence of autonomous oscillations presented in the recent literature [9]–[11], [20]–[22]. Exploiting the interplay between parameter variations and network geometry, we decide to vary those parameters which can affect the topology (adding-removing links).

In the analytical studies of simple two-components networks modelled by differential equations [9]–[11], it was proposed that the presence of a negative feedback loop and high Hill coefficients in the kinetic functions are the key ingredients for the occurrence of oscillatory behaviour. In [20], the authors consider larger systems with three genes, postulating four general requirements for biochemical oscillations: negative feedback, time delay, sufficient non-linearity of the reaction kinetics and proper balance of the timescales of the reactions. In particular, a negative-feedback loop with at least three components can generate oscillations, even without an explicit time delay. It has been further demonstrated that the inclusion of a positive auto-feedback loop can help in obtaining an oscillatory dynamic behaviour [21]. Extending such an idea, in [22] the authors consider topologies in which, in addition to a negative feedback-loop, also a positive one is present, showing that it is generally difficult to adjust a negative feedback oscillators frequency without compromising its amplitude, whereas with positive-plus-negative feedback one can achieve a widely tunable frequency and near-constant amplitude. Thus, positive-plus-negative oscillators appear to be more robust and easier to evolve, rationalizing why they are found in contexts like heartbeats and cell cycles [22].

For the analysis of the IRMA network, we decide to consider only the galactose growing condition, since in such a condition the network is “switched on” and the genes are significantly expressed. Note that, in such condition the protein-protein interaction between Gal4 and Gal80 is switched off (see the section Methods for the details on the mathematical model when only the kinetic parameters). Thus, the topology of IRMA consists of two loops composed only of transcriptional interactions in galactose are considered: one delayed positive feedback loop (DFBL) among the genes *CBF1*, *GAL4*, *SWI5* with a delayed reaction due to the presence of the *HO* promoter (see Methods), and one negative feedback loop (NFBL) among the genes *CBF1*, *GAL4*, *SWI5*, *ASH1* (Figure 1A). The presence of intermediate states in such negative loop suggests that the network has the potentiality of being turned into an autonomous oscillator, if a proper tuning of the parameters is performed.

In what follows, we analyse 3 possible re-engineering scenarios in order both to compare the oscillator tunability and robustness due to different network topologies and to explore different experimental strategies for their implementation.

#### Scenario 1: Stable oscillations keeping the activation of Swi5 on CBF1 (DDEs model). Simulation and continuation results

By looking at the values of the kinetic parameters estimated from *in vivo* data (DDEs model in Methods, parameters in Table 1, Nominal Value column, Figure 1A), it emerges that all the interactions in the NFBL loop are balanced in terms of strength and timescales, except for the maximal velocity of transcription of the *MET16* promoter 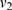 (which drives the expression of *GAL4*) and the Michelis-Menten coefficient 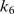, which describes the strength of the activation of *Swi5* on *ASH1* gene. In particular, the parameter 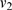 is two order of magnitude lower than all other maximal transcriptional rates while the Michealis-Menten 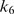 coefficient is one order of magnitude higher. Thus, in order to balance the strength of the regulations involved in the negative feedback loop, we start by decreasing the value of 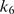 and increasing the value of 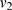, as schematically shown in Figure 1B.

**Table 1:** Parameters of the mathematical models.

Parameter	Nominal Value	Scenario 1 (A, B)	Scenarios 2, 3	Scenario 4 (A, B)
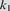 [ 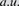 ]	1	1	–	0.0477 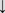
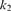 [ 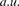 ]	0.035	0.035	0.00035 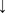	–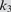 [ 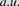 ]	0.037	0.037	0.037	0.037
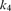 [ 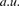 ]	0.010	0.010	0.010	0.010
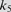 [ 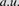 ]	1.884	1.884	1.884	1.884
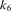 [ 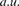 ]	1.884	0.0477 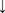	0.0477 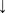	1.884
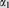 [ 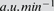 ]	0	0	0	0
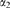 [ 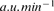 ]	1.49 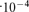	1.49 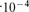	1.49 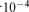	1.49 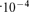
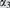 [ 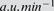 ]	3 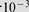	3 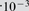	3 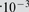	3 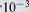
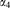 [ 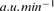 ]	7.4 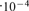	7.4 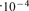	7.4 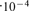	7.4 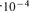
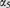 [ 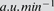 ]	6.1 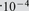	6.1 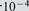	6.1 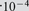	6.1 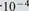
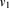 [ 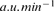 ]	0.040	0.040	0.040	0.040
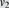 [ 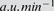 ]	8.82 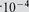	0.026 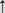 (**A**); 0.001 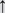 (**B**)	0.026 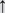	8.82 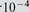 (**B**)
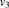 [ 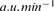 ]	0.020	0.020	0.020	0.020
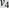 [ 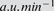 ]	0.014	0.014	0.014	0.014
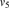 [ 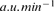 ]	0.018	0.018	0.018	0.018
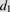 [ 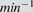 ]	0.022	0.022	0.022	0.022
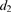 [ 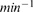 ]	0.047	0.047	0.047	0.047
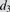 [ 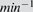 ]	0.421	0.421	0.421	0.421
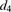 [ 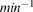 ]	0.098	0.098	0.098	0.098
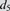 [ 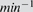 ]	0.050	0.050	0.050	0.050
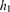	1	1	–	4 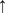
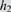	1	4 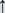	1	–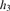	1	1	4 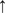	4 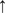 (**A**); 1 (**B**)
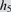	1	1	1	1
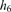	1	4 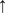	4 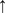	1
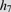	4	4	4	4
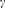 [a.u.]	0.6	0.6	0.6	0.6
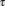 [min]	100	100	100	100

All the parameters are reported. The arrows indicate if the value of the parameter was increased (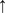) or decreased (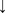) with respect of the nominal value.

**Figure 2:**
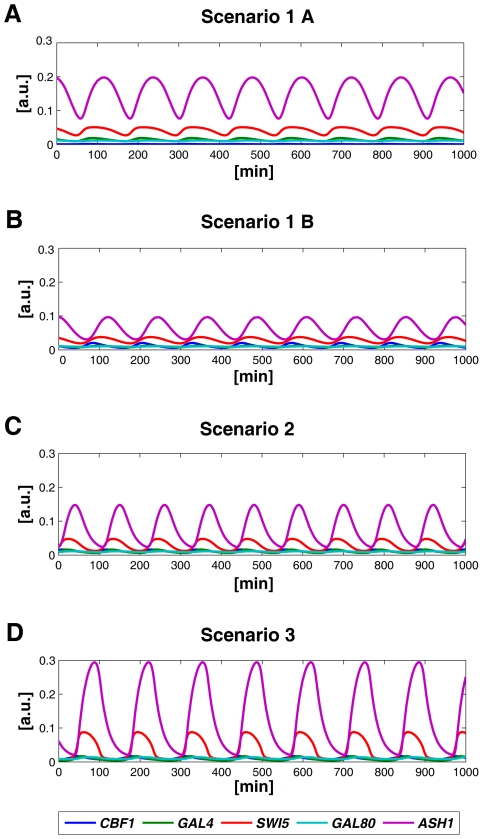
Turning IRMA into an oscillator: time simulations. *In silico* oscillations simulating the mathematical model using the parameters of Scenario 1 A, Scenario 1 B, Scenario 2 and Scenario 3. (A) Scenario 1 A, simulations of the DDEs model; parameters v_2_, k_6_, h_2_ and h_6_ were varied from their nominal values (Table 1, Scenario 1 A column). Period of the oscillations = 120 minutes. (B) Scenario 1 B, simulations of the DDEs model; parameters k_6_, h_2_ and h_6_ were varied from their nominal values like in Scenario 1 A (Table 1, Scenario 1 B column), while v_2_ was tuned according to the continuation results in Figure S1 E in order to increase the values of *CBF1*. Period of the oscillations = 120 minutes. (C) Scenario 2, simulations of the ODEs model; parameters v_2_, k_1_, k_2_, k_6_, h_3_ and h_6_ were varied form their nominal values (Table 1, Scenario 2 column). The negative feedback loop was removed. Period of the oscillations = 110 minutes. (D) Scenario 3, simulations of the ODEs model; parameters v_2_, k_1_, k_2_, k_6_, h_3_ and h_6_ were varied form their nominal values (Table 1, Scenario 3 column). The negative feedback loop was removed. A positive auto-feedback loop was introduced on Swi5. Period of the oscillations = 133 minutes.

Then, we evaluate the effect of the non-linearity of the reaction kinetics generated by the Hill functions on the network behavior. Since the stiffness of such sigmoidal function is determined by the Hill coefficients, which describe the cooperativity of the promoters, we perform our numerical investigation increasing the Hill coefficients 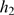 and 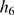 (Figure 1B). According with the parameters choice reported in Table 1 (Scenario 1 A column), the dynamic behaviour of the network appears like in Figure 2A. Here, oscillations have period equal to 120 minutes, thus close to the the yeast cell cycle period in galactose; the amplitude is physically feasible and observable for all the mRNAs, but *CBF1*.

Once oscillations are obtained, a fundamental step in the theoretical analysis is the investigation of the robustness and the tunability of the oscillator. To this aim we use numerical continuation techniques [23]. The transition from a stable steady state solution to a periodic state happens through a supercritical Hopf bifurcation, which occurs when the real part of a complex conjugate pair of eigenvalues of the Jacobian matrix crosses zero, while the real parts of all other eigenvalues remains negative. The software used to perform numerical continuation is DDE-BIFTOOL [3], the first general-purpose package for bifurcation analysis of DDEs. Details on the employed methods and the underlying theory can be found in [24], [25] and in Methods.

The limit cycle can be continued on each of the 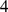 parameters we are varying (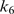, 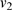, 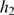, 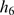). Moreover, once the Hopf bifurcation is localized, it is possible to continue it on all the pairs obtainable by combining such 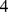 parameters. From continuation results represented in Figure S1, it emerges that keeping the Michelis-Menten parameter 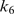 low (i.e. keeping the activation of Swi5 on *ASH1* strong enough) is fundamental to guarantee persistent oscillations. The range of 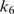 that allows the desired dynamics is further enlarged when the 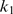 coefficient increases (see Figure S1 A): it means that, if the strength of the positive loop decreases, oscillations are guaranteed only if the strength of the negative loop decreases as well. Figure S1 B shows that 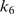 must be kept small if the maximal transcriptional velocity of the *MET16* promoter increases, remarking that the reaction in the loop must be balanced in terms of strength. In Figure S1 C and D we continue the Hopf bifurcation to analyze the relationship between the Hill coefficients 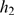 and 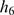 and the Michealis-Menten parameter 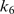, showing that if the activation of Swi5 on *ASH1* is strong enough, the cooperativity coefficient can be decreased without losing persistent oscillations.

Furthermore, continuation allows us to investigate the tunability of the oscillator in terms of amplitude and period (Figure S1 E and F). We found that the amplitude and the period of the oscillations are tunable individually, thus confirming what stated in [22] for topologies that include both a negative and a positive feedback loop. The parameter that was found to affect the period of the oscillations the most is 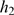: increasing it can enlarge the period up to 18 minutes (Figure S1 F), but the amplitude of the oscillations keeps almost constant (results not shown). Regarding the amplitude, we found that it can be tuned by varying the parameter 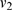 inside the range that ensures oscillations (Figure S1 E). Thus, using continuation we found how to increase the amplitude of *CBF1* oscillations. By simulating the dynamics of the network using the parameters of Scenario 1 B (all parameters identical to Scenario 1 A, but 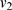 set equal to the value for which the amplitude of 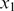 has its maximum in Figure S1 E), we get observable oscillations for all the genes (Figure 2B).

Finally, it is useful to test for the robustness of the oscillator under initial conditions variations. To this aim, we perform a significant number of time simulations (5000) fixing the parameters to the values in Table 1 and changing randomly the initial conditions for all the five genes, keeping all of them into a physical reasonable range ([0 1] [a.u]). The simulations show robustness with all trajectories converging to limit cycles of period 1 (results not shown).

#### Experimental implementation of Scenario 1 *in vivo*

At this point, it is crucial to address the feasibility of re-engineering IRMA *in vivo* according with our theoretical results.

In order to increase the maximal transcription velocity 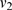 of the *MET16* promoter, the idea is to decrease the level of methionine in the yeast. Methionine modulates the expression of the *MET* genes by affecting the formation of the Cbf1-Met4-Met28 transcriptional complex [26]. High levels of methionine increase the ubiquitination and the subsequent degradation of the activator Met4, indeed inhibiting the transcription [27]. The activation of Cfb1 on Gal4 is the weakest in the actual version of the network, being the *MET16* promoter weak for the methionine concentrations used in our medium (140 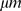) [2].

In Figure S2, we show *in vivo* data (from both semi-quantitative and quantitative real-time RT-PCR) representing the expression levels of the *MET* genes, including *MET16*, when yeast cells are grown in the presence of low (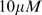) or high (1000 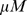) methionine concentration. The levels are compared with the standard yeast growing condition complete medium (YPD), which contains an intermediate concentration of methionine (140 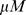) and thus show an intermediate level of *MET* genes expression. *MET16* expression is tightly regulated by methionine concentrations: it is completely turned off in the presence of high methionine levels and, even at intermediate methionine levels (the control condition), its transcription appears to be strongly decreased.

**Figure 3:**
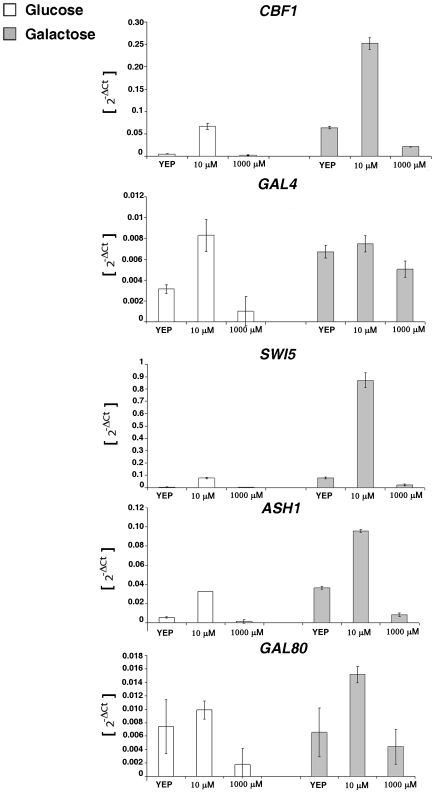
Viability of tuning parameter v_2_ *in vivo*: methionine modulates IRMA genes expression. Expression levels of IRMA genes at different methionine concentrations in glucose (white bars) or in galactose/raffinose (grey bars). The control is the standard complete medium, YEP, which contains 140 mM of methionine. Data represent the 2^−DCt^ (mean of two experiments±Standard Error).

In Figure 3, we show the transcription levels of the genes of IRMA at steady state upon culturing cells in the presence of different concentrations of methionine, both in glucose and in galactose containing medium. Even in the presence of glucose (network off in the control standard growing condition YEP, methionine = 140 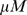), network genes are activated in low methionine containing medium, and reach the same expression levels that they have in the cells grown in galactose (network on in YEP). Thus, the increased *GAL4* expression, due to *MET16* activation after the removal of methionine, turns on all the network genes, while addition of methionine inhibits them, independently from galactose. From such experimental results, we can conclude that increasing the the maximal transcriptional rate 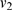, that determines the steady state of the *MET16* promoter and allows to tune the amplitude of the oscillations, can be achieved by simply decreasing the level of methionine in the medium.

Regarding the changes to the *ASH1* promoter, needed to vary 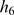 and 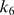, the idea is to replace it with a stronger one. A possible candidate is the *EGT2* promoter [28]. Since this gene is activated even by low levels of Swi5, as well as, by the mutant version of Swi5 (Swi5-AAA) that is present in IRMA [2], it should ensure a low Michaelis-Menten parameter 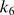, required for obtaining the oscillatory behaviour. Moreover, six putative binding sites have been identified [28], thus ensuring a high Hill coefficient 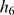.

The last parameter to be tuned is the Hill coefficient 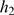. Actually, this would be the most delicate tuning as, in the analyzed scenario, all kinetic parameters of the *HO* promoter are kept equal to their nominal values, but for 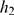, that describes the cooperativity of the inhibition of Ash1 on such promoter. Increasing such cooperativity could be implemented *in vivo* by increasing the number of binding sites for Ash1 on the *HO* promoter, although it has not been previously demonstrated that experimental re-engineering would affect only the Hill coefficient and not other parameters, e.g. the Michaelis-Menten constant of the promoter. Furthermore, such promoter is also activated by Swi5 and the regulatory mechanisms are quite complex [2]. We can conclude that the re-engineering of the *HO* promoter could be troublesome.

#### Scenario 2: Stable oscillations by removing the activation of Swi5 on CBF1 (ODEs model). Simulation and continuation results

The positive loop in Scenario 1 seems difficult to implement in vivo. Therefore, we consider a second scenario, in Figure 1C, in which the delayed activation of Swi5 on Cbf1 is removed and the topology of IRMA is reduced to a negative feedback loop through the genes CBF1, GAL4, SWI5 and ASH1. The corresponding Ordinary Differential Equation model is reporter in Methods.

Again, we tune both the strength of the negative loop (by decreasing 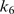 and increasing 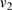) and the non-linearity of the reaction kinetics (by increasing the Hill coefficients 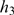 and 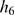). Moreover, we increase the strength of the inhibition of Ash1 on *CBF1* by reducing the value of the Michaelis-Menten coefficient 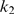. Using the parameters in Table 1 (Scenario 2 column), simulation shows the presence of sustained oscillations with period equal to 110 minutes (Figure 2C). Note that the amplitude of the oscillations is physically feasible and observable for all the genes, including *CBF1*.

Such a scenario can be analyzed in terms of robustness to parameters variations and tunability by using the continuation tool DDE-BIFTOOL with no delayed variable. The most relevant continuation results, reported in Figure S3, lead to conclusions similar to the ones discussed for the first scenario. Namely, it is of utmost importance to keep the Michaelis-Menten parameters 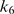 and 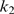 low and the Hill coefficients 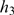 and 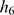 large enough. This confirms that, to have oscillatory behaviour, a proper balance of the reactions in the negative feedback loop is needed together with the presence of significant non-linearities.

Furthermore, through continuation we investigate the tunability of the oscillator, discovering that in Scenario 2, contrary to what found for Scenario 1, it is not possible to tune the amplitude independently of the period. The unique parameter that allows to tune the dynamics of oscillations is 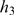, that significantly affects both the period and the amplitude (Figure S3, E and F). Such results confirms what stated in [22] about the tunability of topologies composed only by a negative feedback loop.

Testing through simulations the network dynamics under varying initial conditions within the range [0 1] [a.u], we observe again that robustness is guaranteed. All the trajectories converge to limit cycles of period 1 (results not shown).

#### Experimental implementation of Scenario 2 *in vivo*

The critical parameters which have to be tuned to implement scenario 2 *in vivo* are 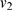, 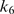, 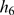, 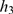, 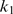 and 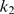. Concerning the first 4, we could proceed like it has been described for Scenario 1: decrease the level of methionine in order to increase the strength of the activation of Cbf1 on Gal4 and replace the *ASH1* promoter with the *EGT2* promoter. Moreover, it is possible to tune also the 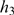 parameter by changing the level of methionine in the yeast. In fact, the behaviour of the *MET16* promoter with low methionine concentrations should become switch like, thus leading to an increase of the stiffness of the sigmoidal Hill function modelled by the 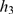 coefficient.

The tuning of parameters 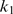 and 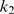 requires two additional changes: first to replace the *HO* promoter with a promoter which is not activated by Swi5. Secondly, we need to replace *ASH1* gene with a gene whose expression is driven by the *EGT2* promoter and that is able to inhibit strongly the new promoter. A good candidate inhibitor-promoter couple is given by *ROX1* repressor and *ANB1* promoter [29].

#### Scenario 3: Stable oscillations by removing the activation of Swi5 on CBF1 and by adding a positive auto-feedback loop on SWI5 (ODEs model). Simulation and continuation results

The topology proposed in Scenario 2 appears feasible for *in vivo* implementation and the oscillations appear robust to varying parameters and initial conditions. For the sake of completeness, we consider also the possibility of including in the network a positive feedback loop, in order to check if the robustness and the tunability of the oscillations increase, according to what shown in a number of works [22], [30], [31].

In Scenario 3, the topology of the network is the same as in Scenario 2 with the addition of an auto-activation reaction on *SWI5* (Figure 1D). The parameters are the same of Scenario 2 (Table 1), but in the Ordinary Differential Equation the equation of *SWI5* needs to be modified, like reported in Methods. Numerical simulations show sustained oscillations with period equal to 133 minutes (Figure 2D). Note that the amplitude of the oscillations is physically feasible and observable for all the genes; in particular, it is significantly higher than in Scenario 2 for the genes *SWI5* and *ASH1*.

We can compare the robustness to parameter variations of Scenarios 2 and 3 by continuing the Hopf bifurcation on the same pairs of parameters considered previously. By comparing Figure S3 A–D and Figure S4 A–D, it appears that the parameter regions that ensure oscillatory behaviour are significantly enlarged. Moreover, unlike the single negative feedback topology, the topology of Scenario 3 allows to tune the amplitude of the oscillations independently from the period (Figure S4, E and F). The period of oscillations can be varied up to 30 minutes, while in Scenario 2 the maximum change was of 10 minutes. Such results confirm that the robustness and the tunability of the network can increase by adding a positive feedback loop.

#### Experimental implementation of Scenario 3 *in vivo*

For the *in vivo* implementation, we need to apply the same changes of Scenario 2 and to add an extra-plasmid containing a strong promoter upstream of the starting codon of *SWI5*. The previously described *EGT2* promoter is again a good candidate.

### Turning IRMA into a Bistable Switch

As our investigation confirms the flexibility of IRMA, we further explore the possibility of turning the network also into a bistable switch. A bistable system is one that toggles between two discrete, alternative stable steady states, in contrast to a monostable system. In biology, bistability has long been established in control of the cell cycle and other oscillations [32], and also recently reported in an artificial gene regulation network [18]. Bistability arises in signaling systems that contain a positive feedback loop or a mutually inhibitory, double negative- feedback loop (which, in some regards, is equivalent to a positive-feedback loop) [33]. Indeed, in [34] it is demonstrated that the existence of at least one positive-feedback loop is is a necessary condition for the existence of multiple steady states.

#### Scenario 4: Continuation results

In our setting, the idea is to reduce the actual version of the topology to a 3 gene positive feedback loop between the genes *CBF1*, *GAL4* and *SWI5*, thus removing the inhibition on *CBF1* by Ash1. The corresponding mathematical model is presented in Methods.

The ODEs model can be analyzed by continuing the steady state on the critical parameters. Figure S5 A and B show typical bistability continuation plots: continuing the steady state on 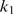 and on 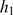 two saddle-node bifurcations delimitate the bistability region in which 3 equilibria coexist, two stable and one unstable. In particular, we can notice that bistability is ensured for 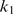 inside the range [0.02 0.14] [a.u.] and 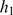 in [2.3 40], thus the activation of Swi5 on Cbf1 must be strong enough. Figure S5 C shows the continuation of one saddle-node bifurcation point on two parameters: a codimension 2 bifurcation point (cusp) is detected, from which two branches delimiting the bistability region for the parameters 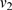 and 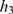 emanate. From such continuation, it emerges that bistability is guaranteed even if we do not vary 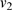 and 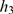 from their nominal values (Table 1, Scenario 4 B column): continuing the steady state on 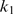, in Figure S5 D we observe again two saddle-node bifurcations delimitating the bistability region that, however, is now slightly smaller ([0.03 0.08] [a.u.]).

#### Experimental implementation of Scenario 4 *in vivo*

For the *in vivo* implementation, a simple strategy is to replace the *HO* promoter by inserting the previously described *EGT2* promoter in front of the *CBF1* gene. Correspondingly, in the model the nominal values of 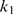 and 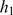 (Michaelis-Menten and Hill coefficient of the *HO* promoter in eq. (7) in Methods) are replaced respectively with 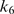 and 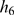. In so doing, the strength and the non-linearity of the positive loop are increased.

Again, we can increase the strength of the activation of the *MET16* promoter by Cbf1 by tuning the parameters 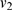 and 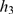 as in the previously analyzed scenarios by decreasing the methionine concentration in the medium. The overall re-engineering of the topology is schematically represented in Figure 1E; the parameters are reported in Table 1, Scenario 4 A column.

## Discussion

In this work, using numerical and continuation techniques, we showed how IRMA can be turned into a robust and tunable oscillator, or a bistable genetic switch. The deterministic mathematical model, previously formulated and identified to allow data interpretation and experiment planning, is here analysed to guide the re-engineering of the network with predictable functions.

IRMA showed great flexibility. Its topology can be re-engineered in a number of ways in order to achieve the desired dynamical behaviour. Of note, all the proposed changes are viable *in vivo*. The robustness to parameters changes and the tunability of the oscillator were assessed via continuations performed using the software DDE-BIFTOOL, the first package for bifurcation analysis of systems with delays that, up to now, has not been commonly used in the Synthetic Biology community.

The major conclusion we can draw from our results is that, aiming at constructing a robust and tunable oscillator, the best option is to include in the topology both a delayed negative feedback loop and a fast positive one. This is the case explicitly analyzed in Scenario 3 that results to be most robust and tunable as compared to Scenario 2, in which the topology of the network is reduced to a single negative feedback loop.

In the context of Synthetic Biology, our model guided re-engineering framework can be applied to existing topologies with the aim of turning them into oscillators or switches. We analyzed three topologies for the oscillator case and one for the switch case. A crucial point was to minimize the number of experiments needed to modify the synthetic network. Surely, other possible ways to re-engineering IRMA can give rise to other oscillatory, switch-like and maybe more complex dynamical behaviours. Of note, once the best performing scenario has been chosen from our deterministic approach, it will be crucial to resort to stochastic simulations in order to estimate the impact of noise on the network dynamics [35]. Remarkably, resulting noise-induced bifurcations can lead to multi-stability or oscillatory dynamics in biochemical networks even when the deterministic description predicts a stable steady state for a certain parameter set [36], or for any parameter values [37].

## Methods

### Mathematical Models

The mathematical model we used is made up of of five nonlinear Delay Differential Equations that describe the production rates of the five mRNA concentrations, assuming Hill kinetics and proportionality between protein and mRNA levels. The time delay, describing the delayed activation of the *HO* promoter by Swi5, is fixed (100 minutes). In [2] the model was identified and extensively validated against experimental data. The 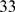 unknown parameters (3 parameters are medium dependent) were estimated from time series data. In order to fit the Glucose to Galactose time-series, we further included a transient term in the degradations of *GAL4* and *GAL80* that describes the starvation effect due to the washing of the cells before the medium shift [2].

For this work, the medium-dependent parameters in the equation of *SWI5* are fixed to their values in galactose. Moreover, since the transient dynamics are neglected, the starvation effect induced by the medium shift is removed.

Letting 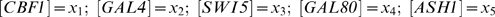, the model is:
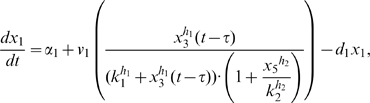
(1)

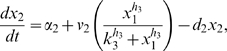
(2)

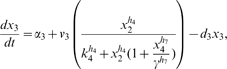
(3)

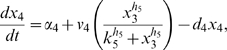
(4)

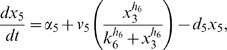
(5)where 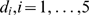 are the degradation rates, 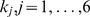 are the Michaelis-Menten constants, 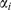 are the basal activities, 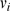 represent the maximal transcription rates. 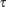 models the delay in the *HO* promoter. Since the real-time qPCR data showed in [2] were obtained as ratio of fluorescence normalized with respect to *ACT1* gene, concentrations are reported in arbitrary units [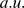], the degradation rates 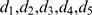 in [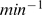], the Michaelis-Menten constants 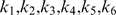 in [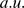], the affinity constant 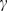 in [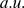], the basal activities 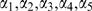 in [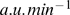], the maximal transcription rates in [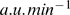]. In Table 1 (Nominal values column) the estimated parameters are reported.

The model analyzed in **Scenario 1** is the DDEs model composed of equations (1)–(5).

To describe **Scenario 2**, the positive feedback loop on *CBF1* is removed. In the model, this corresponds to fixing the Michealis-Menten coefficient 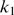 to zero or equivalently rewrite equation (1) as:
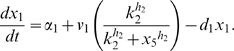
(6)


To describe **Scenario 3**, the positive feedback loop on *CBF1* is removed and a positive auto-feedback loop on *SWI5* is inserted. The auto-activation is driven by the same promoter that drives the expression of *ASH1*. In the ODEs model, these changes correspond to fixing the Michealis-Menten coefficient 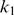 to zero, thus substituting (1) with (6), and adding an activation term in equation (3) that becomes:
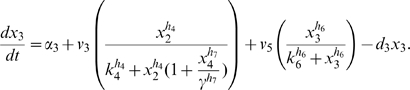
(7)


To obtain **Scenario 4**, the negative feedback loop is removed, and the positive one on *CBF1* is not delayed. The ODEs model consists of equations (2)–(5) while equation (1) is replaced with:
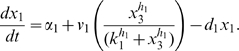
(8)


### Numerical Simulations and Continuations

Numerical simulations were run using Matlab 2008b (The MathWorks). For Scenario 1, we adopted the *dde23* solver, which solves delay differential equations (DDEs) with constant delays (a detailed discussion of the numerical method can be found in [38]). In the case of Scenario 2, 3 and 4 (ODE models), we used the *ode23* solver (a detailed discussion of the numerical methods on which *ode23* relies can be found in [39]).

All the numerical continuation experiments were performed using DDE-BIFTOOL package [3]. The characteristic matrix appearing in the stability theory for DDEs has an infinite number of eigenvalues because of the infinite-dimensional nature of DDEs. To determinate the local stability of an equilibrium, in DDE-BIFTOOL [3] a linear multi-step method is applied to the variational equation and the approximations to the rightmost (stability determining) characteristic roots are computed. In case of periodic solution of period *T*, a discrete approximation on a mesh in [0, *T*] and its period are computed as solutions of the corresponding periodic boundary value problem by using a piecewise polynomial collocation. The local asymptotic stability of a periodic solution is determined by the spectrum of the linear so-called monodromy operator [3].

## Supporting Information

Figure S1 Continuation results for Scenario 1 A using DDE-BIFTOOL software. (A) Two parameters continuation of the Hopf bifurcation on parameters k_1_ (Michealis-Menten coefficient of the *HO* promoter) and k_6_ (Michealis-Menten coefficient of the *ASH1* promoter). (B) Two parameters continuation of the Hopf bifurcation on parameters v_2_ (maximal transcriptional rate of the *MET16* promoter) and k_6_ (Michealis-Menten coefficient of the *ASH1* promoter). (C) Two parameters continuation of the Hopf bifurcation on parameters k_6_ (Michealis-Menten coefficient of the *ASH1* promoter) and h_2_ (Hill coefficient of the *HO* promoter). (D) Two parameters continuation of the Hopf bifurcation on parameters h_2_ (Hill coefficient of the *HO* promoter) and h_6_ (Hill coefficient of the *ASH1* promoter). (E) Tunability of the oscillations in terms of amplitude. Amplitude of x_1_ (level of the *CBF1* gene) continuing the periodic solution on v_2_ (maximal transcriptional rate of the *MET16* promoter). (F) Tunability of the oscillations in terms of period. Period of x_1_ (*CBF1* gene) continuing the periodic solution on h_2_ (Hill coefficient of the *HO* promoter).
(0.26 MB TIF)

Figure S2 Expression of *MET* genes in wild type yeast cells. *MET* genes regulated by Cbf1 are transcriptionally activated in the presence of low levels of methionine (10 µm) while they are repressed at high methionine concentrations (1000 µm). Semi-quantitative (A) and quantitative (B) RT-PCR (normalization against *ACT1* gene)of *MET* genes were performed on total RNA extracted from yeast cells grown in the standard complete medium YPD (140 µm of methionine)and at two different methionine concentrations.
(0.47 MB TIF)

Figure S3 Continuation results for Scenario 2. Continuation results for Scenario 2 using DDE-BIFTOOL software. (A) Two parameters continuation of the Hopf bifurcation on parameters k_2_ (Michealis-Menten coefficient of the *HO* promoter) and h_3_ (Hill coefficient of the *MET16* promoter). (B) Two parameters continuation of the Hopf bifurcation on parameters v_2_ (maximal transcriptional rate of the *MET16* promoter) and k_6_ (Michealis-Menten coefficient of the *ASH1* promoter). (C) Two parameters continuation of the Hopf bifurcation on parameters k_6_ (Michealis-Menten coefficient of the *ASH1* promoter) and h_3_ (Hill coefficient of the *MET16* promoter). (D) Two parameters continuation of the Hopf bifurcation on parameters h_3_ (Hill coefficient of the *MET16* promoter) and h_6_ (Hill coefficient of the *ASH1* promoter). (E) Tunability of the oscillations in terms of amplitude. Amplitude of x_1_ (level of the *CBF1* gene) continuing the periodic solution on h_3_ (Hill coefficient of the *MET16* promoter). (F) Tunability of the oscillations in terms of period. Period of x_1_ (*CBF1* gene) continuing the periodic solution on h_3_ (Hill coefficient of the *MET16* promoter).
(0.30 MB TIF)

Figure S4 Continuation results for Scenario 3. Continuation results for Scenario 3 using DDE-BIFTOOL software. (A) Two parameters continuation of the Hopf bifurcation on parameters k_2_ (Michealis-Menten coefficient of the *HO* promoter) and h_3_ (Hill coefficient of the *MET16* promoter). (B) Two parameters continuation of the Hopf bifurcation on parameters v_2_ (maximal transcriptional rate of the *MET16* promoter) and k_6_ (Michealis-Menten coefficient of the *ASH1* promoter). (C) Two parameters continuation of the Hopf bifurcation on parameters k_6_ (Michealis-Menten coefficient of the *ASH1* promoter) and h_3_ (Hill coefficient of the *MET16* promoter). (D) Two parameters continuation of the Hopf bifurcation on parameters h_3_ (Hill coefficient of the *MET16* promoter) and h_6_ (Hill coefficient of the _ASH1_ promoter). (E) Tunability of the oscillations in terms of amplitude. Amplitude of x_1_ (level of the *CBF1* gene) continuing the periodic solution on h_3_ (Hill coefficient of the *MET16* promoter). (F) Tunability of the oscillations in terms of period. Period of x_1_ (*CBF1* gene) continuing the periodic solution on h_6_ (Hill coefficient of the *ASH1* promoter).
(0.26 MB TIF)

Figure S5 Continuation results for Scenario 4. (A) Scenario 4 A. One parameter continuation of the steady state on k_1_ (Michealis-Menten coefficient of the *HO* promoter). Two saddle-node bifurcation points (at (k_1_; x_1_) = (0.02 0.007) and (k_1_; x_1_) = (0.14 0.01)) delimitate the bistability region. (B) Scenario 4 A. One parameter continuation of the steady state on h_1_ (Hill coefficient of the *HO* promoter). Two saddle-node bifurcations (at (h_1_; x_1_) = (2 0.008) and (h_1_; x_1_) = (40 0.019)) delimitate the bistability region. (C) Scenario 4 A. Two parameters continuation of one saddle-node bifurcation point on v_2_ (maximal transcriptional rate of the *MET16* promoter) and h_3_ (Hill coefficient of the *MET16* promoter). The cusp bifurcation occurs at (v_2_; h_3_) = (0.0005 0.39). (D) Scenario 4 B. One parameter continuation of the steady state on k_1_ (Michealis-Menten coefficient of the *HO* promoter). Two saddle-node bifurcation points (at (k_1_; x_1_) = (0.03 0.002) and (k_1_; x_1_) = (0.08 0.05)) delimitate the bistability region.
(0.27 MB TIF)
