# Supplementary figures and images for: How to Turn a Genetic Circuit into a Synthetic Tunable Oscillator, or a Bistable Switch
Source: PLoS One. 2009 Dec 7;4(12):e8083. doi: 10.1371/journal.pone.0008083 (PMC2784219; doi:10.1371/journal.pone.0008083)

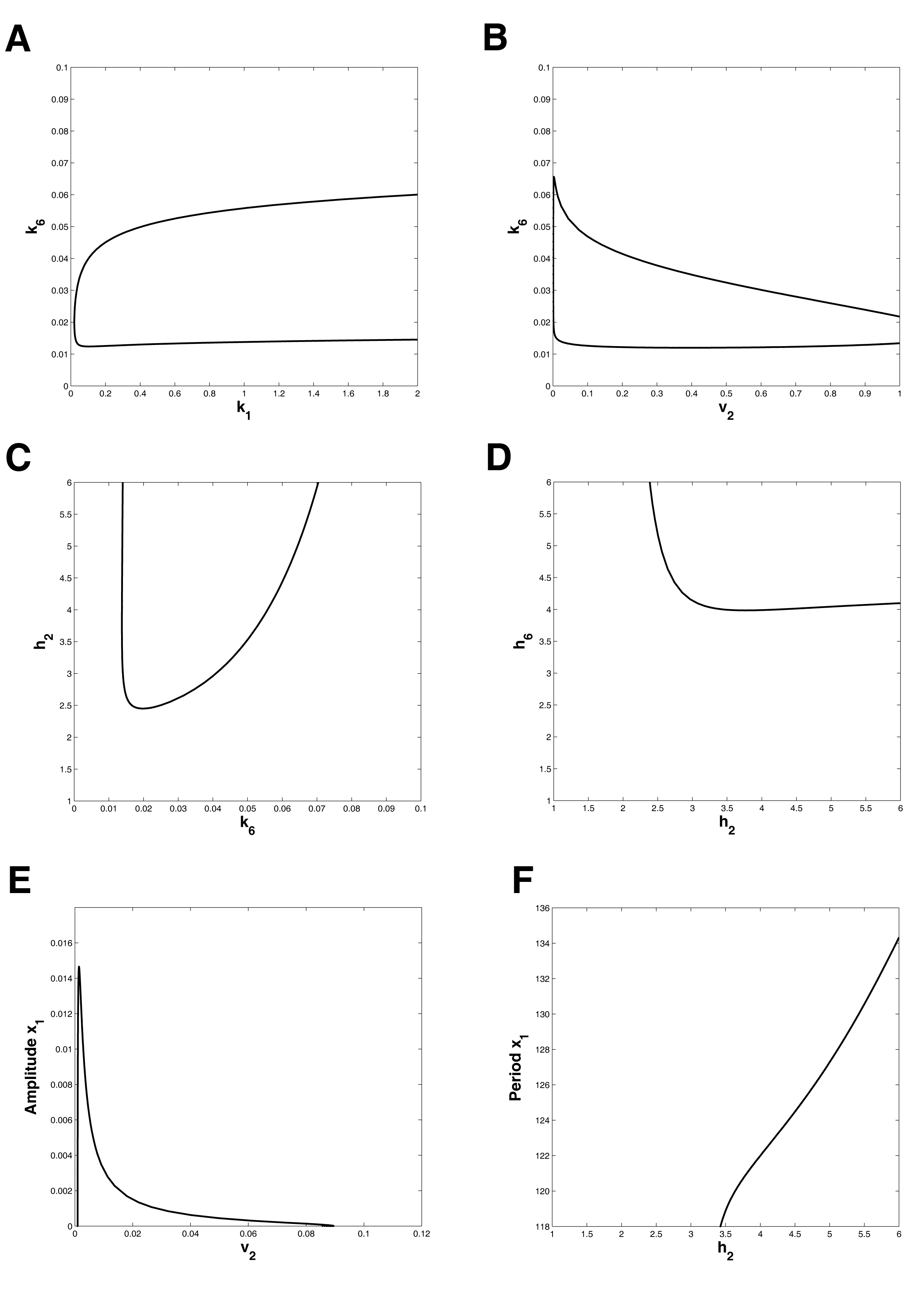

Supplement: Figure S1 — Continuation results for Scenario 1 A using DDE-BIFTOOL software. (A) Two parameters continuation of the Hopf bifurcation on parameters k1 (Michealis-Menten coefficient of the HO promoter) and k6 (Michealis-Menten coefficient of the ASH1 promoter). (B) Two parameters continuation of the Hopf bifurcation on parameters v2 (maximal transcriptional rate of the MET16 promoter) and k6 (Michealis-Menten coefficient of the ASH1 promoter). (C) Two parameters continuation of the Hopf bifurcation on parameters k6 (Michealis-Menten coefficient of the ASH1 promoter) and h2 (Hill coefficient of the HO promoter). (D) Two parameters continuation of the Hopf bifurcation on parameters h2 (Hill coefficient of the HO promoter) and h6 (Hill coefficient of the ASH1 promoter). (E) Tunability of the oscillations in terms of amplitude. Amplitude of x1 (level of the CBF1 gene) continuing the periodic solution on v2 (maximal transcriptional rate of the MET16 promoter). (F) Tunability of the oscillations in terms of period. Period of x1 (CBF1 gene) continuing the periodic solution on h2 (Hill coefficient of the HO promoter). (0.26 MB TIF) [file pone.0008083.s001.tif]

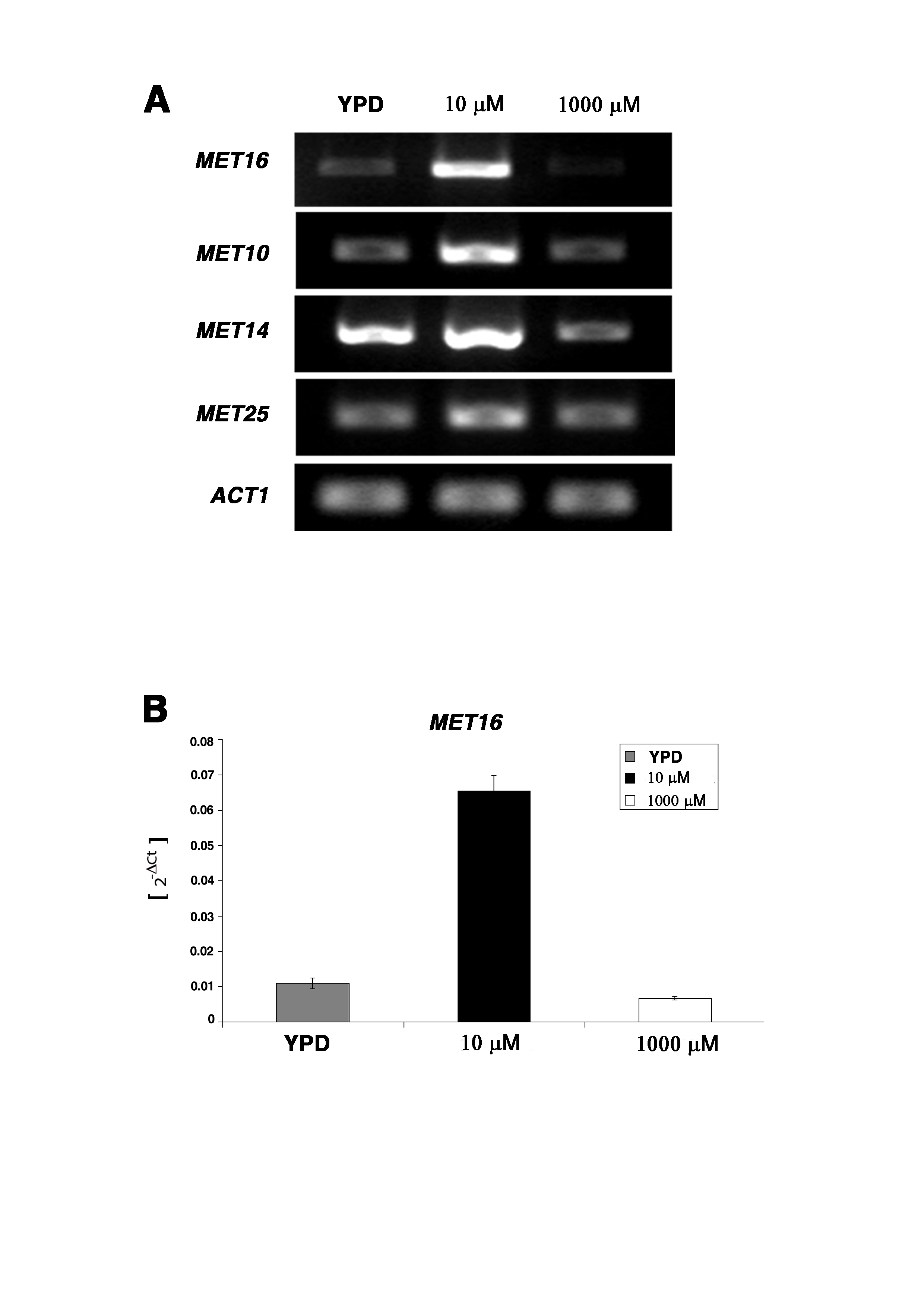

Supplement: Figure S2 — Expression of MET genes in wild type yeast cells. MET genes regulated by Cbf1 are transcriptionally activated in the presence of low levels of methionine (10 µm) while they are repressed at high methionine concentrations (1000 µm). Semi-quantitative (A) and quantitative (B) RT-PCR (normalization against ACT1 gene)of MET genes were performed on total RNA extracted from yeast cells grown in the standard complete medium YPD (140 µm of methionine)and at two different methionine concentrations. (0.47 MB TIF) [file pone.0008083.s002.tif]

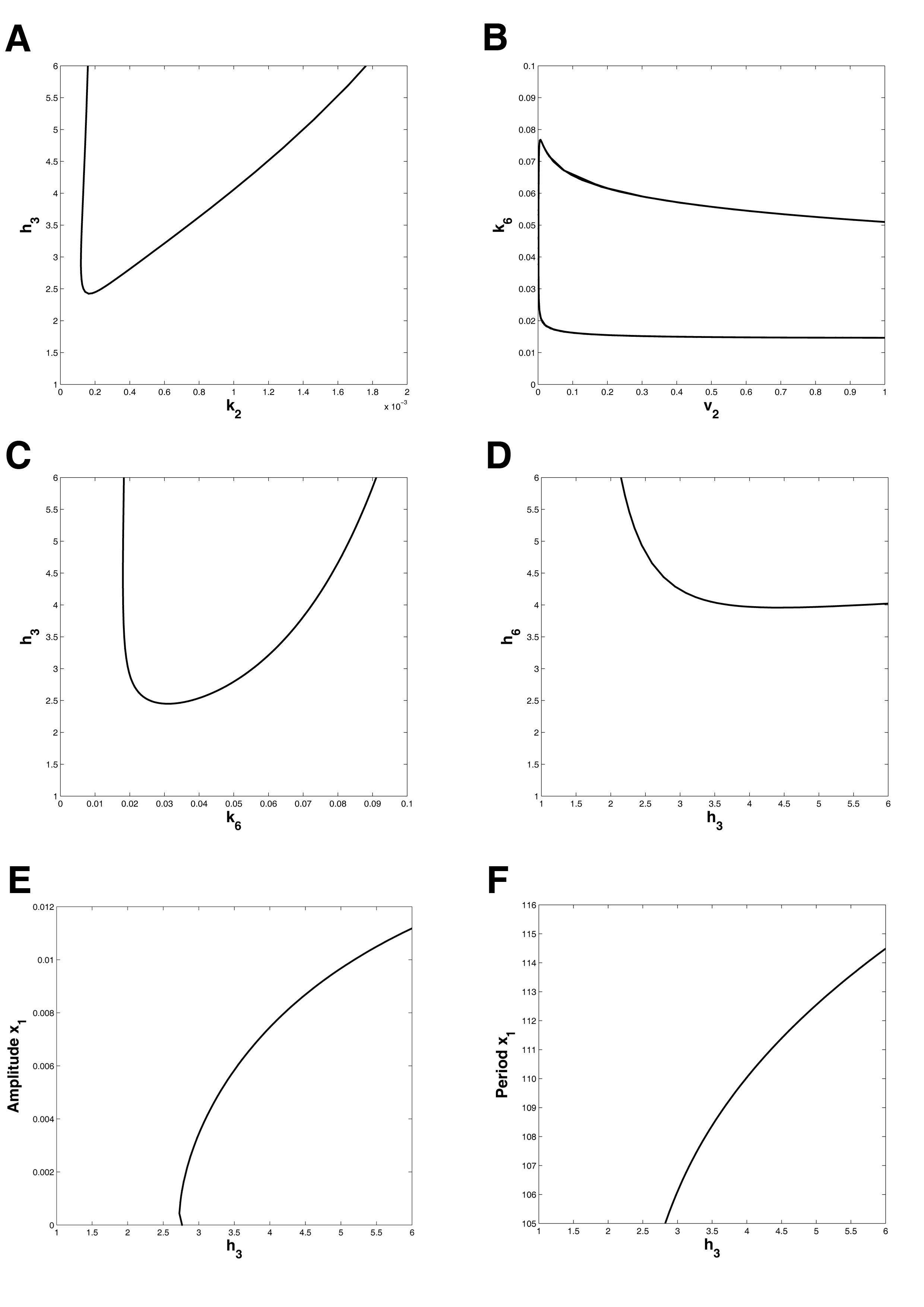

Supplement: Figure S3 — Continuation results for Scenario 2. Continuation results for Scenario 2 using DDE-BIFTOOL software. (A) Two parameters continuation of the Hopf bifurcation on parameters k2 (Michealis-Menten coefficient of the HO promoter) and h3 (Hill coefficient of the MET16 promoter). (B) Two parameters continuation of the Hopf bifurcation on parameters v2 (maximal transcriptional rate of the MET16 promoter) and k6 (Michealis-Menten coefficient of the ASH1 promoter). (C) Two parameters continuation of the Hopf bifurcation on parameters k6 (Michealis-Menten coefficient of the ASH1 promoter) and h3 (Hill coefficient of the MET16 promoter). (D) Two parameters continuation of the Hopf bifurcation on parameters h3 (Hill coefficient of the MET16 promoter) and h6 (Hill coefficient of the ASH1 promoter). (E) Tunability of the oscillations in terms of amplitude. Amplitude of x1 (level of the CBF1 gene) continuing the periodic solution on h3 (Hill coefficient of the MET16 promoter). (F) Tunability of the oscillations in terms of period. Period of x1 (CBF1 gene) continuing the periodic solution on h3 (Hill coefficient of the MET16 promoter). (0.30 MB TIF) [file pone.0008083.s003.tif]

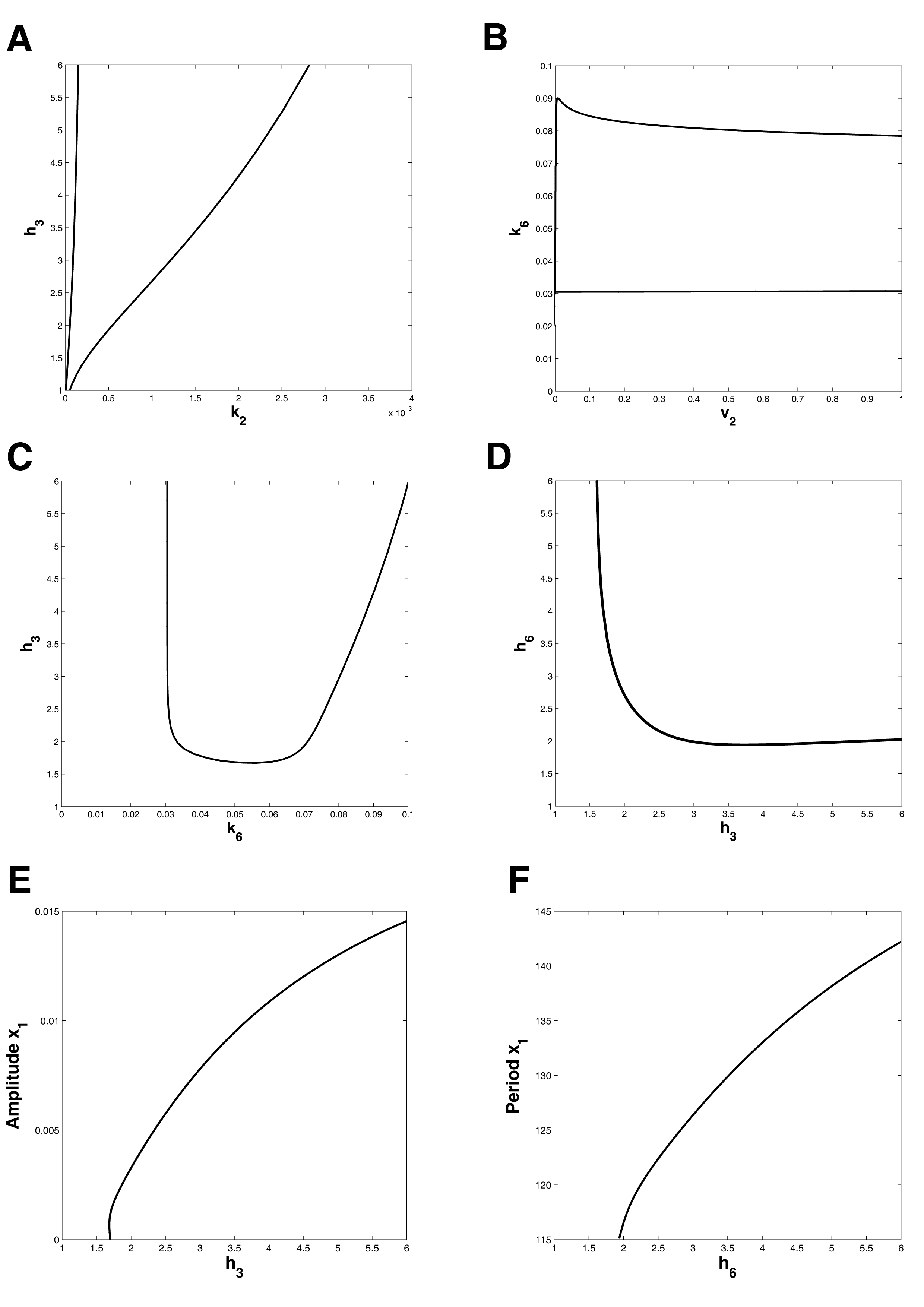

Supplement: Figure S4 — Continuation results for Scenario 3. Continuation results for Scenario 3 using DDE-BIFTOOL software. (A) Two parameters continuation of the Hopf bifurcation on parameters k2 (Michealis-Menten coefficient of the HO promoter) and h3 (Hill coefficient of the MET16 promoter). (B) Two parameters continuation of the Hopf bifurcation on parameters v2 (maximal transcriptional rate of the MET16 promoter) and k6 (Michealis-Menten coefficient of the ASH1 promoter). (C) Two parameters continuation of the Hopf bifurcation on parameters k6 (Michealis-Menten coefficient of the ASH1 promoter) and h3 (Hill coefficient of the MET16 promoter). (D) Two parameters continuation of the Hopf bifurcation on parameters h3 (Hill coefficient of the MET16 promoter) and h6 (Hill coefficient of the ASH1 promoter). (E) Tunability of the oscillations in terms of amplitude. Amplitude of x1 (level of the CBF1 gene) continuing the periodic solution on h3 (Hill coefficient of the MET16 promoter). (F) Tunability of the oscillations in terms of period. Period of x1 (CBF1 gene) continuing the periodic solution on h6 (Hill coefficient of the ASH1 promoter). (0.26 MB TIF) [file pone.0008083.s004.tif]

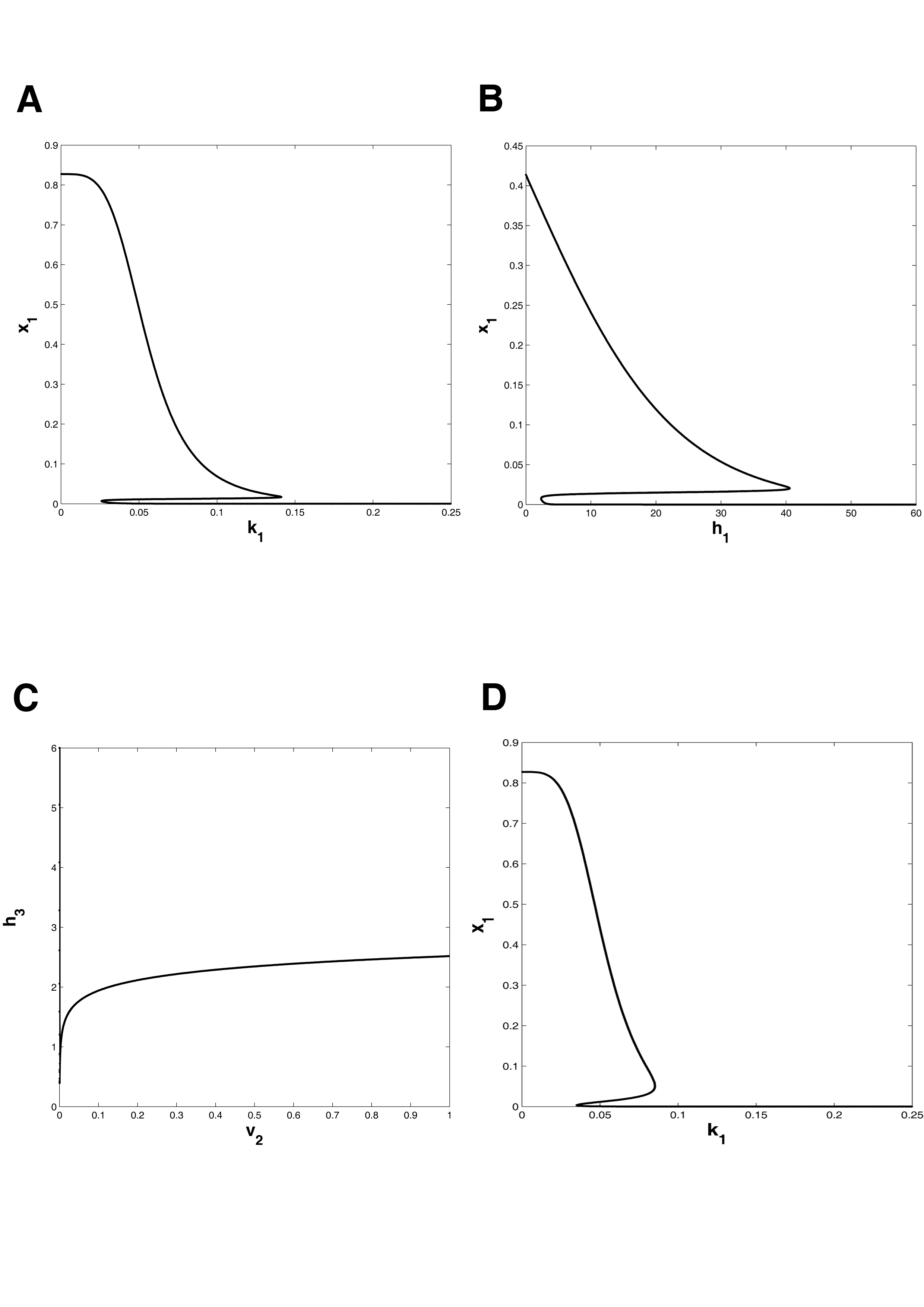

Supplement: Figure S5 — Continuation results for Scenario 4. (A) Scenario 4 A. One parameter continuation of the steady state on k1 (Michealis-Menten coefficient of the HO promoter). Two saddle-node bifurcation points (at (k1; x1) = (0.02 0.007) and (k1; x1) = (0.14 0.01)) delimitate the bistability region. (B) Scenario 4 A. One parameter continuation of the steady state on h1 (Hill coefficient of the HO promoter). Two saddle-node bifurcations (at (h1; x1) = (2 0.008) and (h1; x1) = (40 0.019)) delimitate the bistability region. (C) Scenario 4 A. Two parameters continuation of one saddle-node bifurcation point on v2 (maximal transcriptional rate of the MET16 promoter) and h3 (Hill coefficient of the MET16 promoter). The cusp bifurcation occurs at (v2; h3) = (0.0005 0.39). (D) Scenario 4 B. One parameter continuation of the steady state on k1 (Michealis-Menten coefficient of the HO promoter). Two saddle-node bifurcation points (at (k1; x1) = (0.03 0.002) and (k1; x1) = (0.08 0.05)) delimitate the bistability region. (0.27 MB TIF) [file pone.0008083.s005.tif]
